# Human-robot cooperative movement training: Learning a novel sensory motor transformation during walking with robotic assistance-as-needed

**DOI:** 10.1186/1743-0003-4-8

**Published:** 2007-03-28

**Authors:** Jeremy L Emken, Raul Benitez, David J Reinkensmeyer

**Affiliations:** 1 Biomedical Engineering Department, University of California at Irvine, Irvine, CA, USA; 2 Mechanical and Aerospace Engineering Department, University of California at Irvine, Irvine, CA, USA; 3 Automatic Control Department, Universitat Politècnica de Catalunya, Barcelona, SPAIN

## Abstract

**Background:**

A prevailing paradigm of physical rehabilitation following neurologic injury is to "assist-as-needed" in completing desired movements. Several research groups are attempting to automate this principle with robotic movement training devices and patient cooperative algorithms that encourage voluntary participation. These attempts are currently not based on computational models of motor learning.

**Methods:**

Here we assume that motor recovery from a neurologic injury can be modelled as a process of learning a novel sensory motor transformation, which allows us to study a simplified experimental protocol amenable to mathematical description. Specifically, we use a robotic force field paradigm to impose a virtual impairment on the left leg of unimpaired subjects walking on a treadmill. We then derive an "assist-as-needed" robotic training algorithm to help subjects overcome the virtual impairment and walk normally. The problem is posed as an optimization of performance error and robotic assistance. The optimal robotic movement trainer becomes an error-based controller with a forgetting factor that bounds kinematic errors while systematically reducing its assistance when those errors are small. As humans have a natural range of movement variability, we introduce an error weighting function that causes the robotic trainer to disregard this variability.

**Results:**

We experimentally validated the controller with ten unimpaired subjects by demonstrating how it helped the subjects learn the novel sensory motor transformation necessary to counteract the virtual impairment, while also preventing them from experiencing large kinematic errors. The addition of the error weighting function allowed the robot assistance to fade to zero even though the subjects' movements were variable. We also show that in order to assist-as-needed, the robot must relax its assistance at a rate faster than that of the learning human.

**Conclusion:**

The assist-as-needed algorithm proposed here can limit error during the learning of a dynamic motor task. The algorithm encourages learning by decreasing its assistance as a function of the ongoing progression of movement error. This type of algorithm is well suited for helping people learn dynamic tasks for which large kinematic errors are dangerous or discouraging, and thus may prove useful for robot-assisted movement training of walking or reaching following neurologic injury.

## Background

Robot-assisted movement training following neurologic injury is a promising new field that seeks to automate hands-on therapy and promote neural recovery [1-4]. Currently, however, it is unclear how robots should assist in therapy in order to best promote neural recovery. Experienced rehabilitation therapists advocate "active assist exercise" or "assisting as needed", which refers to the principle of helping a patient perform a movement with the minimal amount of manual assistance possible [5].

Several robot control strategies have been designed to aid in active assist exercise following neurological injury, for both upper extremity and gait training. [1,6-11]. Mechanical guidance of the affected limb through a predetermined trajectory is the predominant training method in the arms during reaching tasks [1,8,12-14] and the legs during walking on a treadmill [9,11], although force-based techniques that increase the patient's effort [8,15] or amplify subject errors have also been proposed [7,16].

Recent efforts to improve the performance of the widely-used MIT-MANUS device have focused on making the device interactive by allowing EMG activity in selected muscles to trigger robotic assistance to complete movements in the horizontal plane [17], or by adjusting the robot assistance based on metrics of patient performance [18]. For locomotion training with the Lokomat device, robotic assistance is also being designed to be "patient cooperative" [6]. For example, algorithms that adjust the desired movement trajectory and impedance of the robot based on the robot-subject interaction force are in development, and visual biofeedback displays are being developed to inform patients of their contribution to their imposed movement [19]. However, although cleverly designed, these algorithms are currently unsupported by rigorous modelling of the way that the human motor system adapts. Developing algorithms based on an understanding of the neural computations involved in adaptive control could provide a theoretical foundation for appropriate control strategies, and help direct clinical testing.

In this paper, we assume that the recovery process following a neurologic injury can be modelled as the learning of a novel sensory motor transformation. In other words, following a neurologic injury, the human motor system must re-learn the correct spatio-temporal pattern of muscle activation to achieve a desired limb trajectory. To facilitate computational analysis of this process, we study a simplified experimental protocol in this paper. Specifically, we use a robotic force field paradigm [20] to impose a virtual impairment on the left leg of unimpaired subjects walking on a treadmill. This virtual impairment perturbs the natural walking pattern, and requires the subjects to learn a novel sensory motor transformation in order to walk normally again. Thus, this protocol captures a process that is computationally similar to a key process involved in movement training following neurologic injury – i.e. the learning of new sensory motor transformation. In addition, the protocol is much more readily implemented than labor- and time-intensive clinical rehabilitation, and more amenable to quantitative analysis. However, the protocol studied here is not rehabilitation, and thus represents at best a "starting framework" for deriving rigorous robot training strategies for rehabilitation.

The key question this paper addresses is: "How can a robot best assist a person in learning a novel sensory motor transformation while limiting kinematic errors?" We formulate this "assist-as-needed" principle as an optimization problem. We assume that the robotic movement trainer must minimize a cost function that is the weighted sum of robot force and subject movement error as the subject learns a novel sensory motor transformation. We use an experimentally validated, computational model of internal model formation [21] that uses the perturbing force and previous kinematic error to predict the future value of that error. The resulting control law allows motor learning while constraining kinematic error, and systematically reduces its assistance as learning progresses. Here we experimentally validate the use of this controller and test a fundamental prediction that in order to assist-as-needed, the robot must relax its assistance at a rate faster than the human motor system learns to decrement its own force. That is, the robot must adapt its performance to the learning human faster than the human adapts to the novel sensory motor perturbation. This allows the robot to stay one-step ahead of the human, always challenging and not allowing the human to come to rely on it.

## Methods

### Creating a Virtual Impairment for a Walking Task

To provide a context for the following controller derivation, assume that we are interested in designing a robotic control law for step training on a treadmill. We would like the robotic device to assist in re-training the swing phase of gait in the presence of an impairment that disrupts the kinematics of leg swing. In this paper, we use the robotic device to create a virtual impairment that is applied to unimpaired subjects as they walk on a treadmill. The virtual impairment is arbitrarily chosen as a force that is applied only during the swing phase of gait, and that pushes the leg upward with a force proportional to the forward velocity of the subject's ankle. Thus, the virtual impairment tends to make the subject step with an abnormally high step trajectory during swing. When an unimpaired person is exposed to such a virtual impairment, they will learn to adapt to it over the course of tens of steps by learning how to anticipate the perturbing forces; that is, by learning a new sensory motor transformation between the desired step trajectory and the required muscle activations [16].

### Assistance-as-needed as an optimization problem

We quantify motor performance for this task by step height x_i _on the i^th ^step, and robot performance by the upward force R_i _exerted on the ankle on the i^th ^step. We would like to design a robotic movement trainer that allows the subject to learn how to overcome the virtual impairment, but that also limits kinematic error experienced during this learning process. We therefore require that the robotic movement trainer minimize a weighted sum of error and assistance force:

J=12(xi+1−xf)2+λR2(Ri+1)2     (1)
 MathType@MTEF@5@5@+=feaafiart1ev1aaatCvAUfKttLearuWrP9MDH5MBPbIqV92AaeXatLxBI9gBaebbnrfifHhDYfgasaacH8akY=wiFfYdH8Gipec8Eeeu0xXdbba9frFj0=OqFfea0dXdd9vqai=hGuQ8kuc9pgc9s8qqaq=dirpe0xb9q8qiLsFr0=vr0=vr0dc8meaabaqaciaacaGaaeqabaqabeGadaaakeaacqWGkbGscqGH9aqpdaWcaaqaaiabigdaXaqaaiabikdaYaaacqGGOaakcqWG4baEdaWgaaWcbaGaemyAaKMaey4kaSIaeGymaedabeaakiabgkHiTiabdIha4naaBaaaleaacqWGMbGzaeqaaOGaeiykaKYaaWbaaSqabeaacqaIYaGmaaGccqGHRaWkdaWcaaqaaiabeU7aSnaaBaaaleaacqWGsbGuaeqaaaGcbaGaeGOmaidaaiabcIcaOiabdkfasnaaBaaaleaacqWGPbqAcqGHRaWkcqaIXaqmaeqaaOGaeiykaKYaaWbaaSqabeaacqaIYaGmaaGccaWLjaGaaCzcamaabmaabaGaeGymaedacaGLOaGaayzkaaaaaa@4C8C@

where x_f _is the desired step height in the field and λ_R _is a constant which weights the relative cost of the error and force terms. Notice that minimizing this cost function requires satisfying two competing goals: applying as little force as possible and making the person step as close to the normative step height, x_f_, as possible. Thus, this cost function formalizes the principle of "assist-as-needed".

In order to find the controller that minimizes this cost function, we must model how the leg responds to applied forces. We assume that the subject adapts to a perturbing force field, F_i _applied to the leg on the i^th ^step with the following dynamics [16,22,23]:

*e*_*i*+1 _= *a*_0_*e*_*i *_+ *b*_1_*F*_*i *_+ *b*_0_*F*_*i*+1_,     (2)

where *e*_*i *_= *x*_*i *_- *x*_*d *_is the kinematic error during the i^th ^step, and F_i _is in the form of a perpendicularly directed viscous force field applied only during the swing phase of gait. We quantify step height x_i _on the i^th ^step at 300 ms following initial forward motion of the ankle during swing (i.e. approximately at mid-swing), and the robot force field R_i _as the force exerted on the ankle on the i^th ^step 100 ms following initial forward motion of the ankle (i.e. early in swing). It can be shown that these parameter values maximize the fit of equation 2 to the experimental data, although other measures such as peak step height and peak field strength will also suffice [16]. Note for the case studied here of subjects adapting to an external force field, x_d _is the step height during stepping with no applied field and x_f _is the steady state step height following adaptation to the force field. Thus, x_f _is the desired step height in the applied field.

The dynamics in equation 2 capture the process of internal model formation, which has been quantified in several experiments examining motor adaptation to imposed novel dynamic environments [21-23]. We have shown elsewhere that these dynamics minimize a cost function containing error, effort, and change in effort terms [21]. Further, they can be viewed as arising from the interaction of spring-like leg dynamics with the following muscle controller:

*u*_*i*+1 _= *f*_*H*_*u*_*i *_- *g*_*H*_*e*_*i*_,     (3)

where u_i _is force from muscular activity on the i^th ^movement trial, f_H _< 1 is a human forgetting factor, and g_H _is the motor system's feedback gain for error-based correction of the muscle activity. Thus, our basic assumption about how the nervous system responds to an applied force is that it tries to model the force then counteract it, using an error-based learning controller, on a movement-by-movement basis. The parameters of equation 2 are related to the parameters of the controller as follows:

a0=fH−gHKb1=−fHKb0=1K     (4)
 MathType@MTEF@5@5@+=feaafiart1ev1aaatCvAUfKttLearuWrP9MDH5MBPbIqV92AaeXatLxBI9gBaebbnrfifHhDYfgasaacH8akY=wiFfYdH8Gipec8Eeeu0xXdbba9frFj0=OqFfea0dXdd9vqai=hGuQ8kuc9pgc9s8qqaq=dirpe0xb9q8qiLsFr0=vr0=vr0dc8meaabaqaciaacaGaaeqabaqabeGadaaakeaafaqabeqadaaabaGaemyyae2aaSbaaSqaaiabicdaWaqabaGccqGH9aqpcqWGMbGzdaWgaaWcbaGaemisaGeabeaakiabgkHiTmaalaaabaGaem4zaC2aaSbaaSqaaiabdIeaibqabaaakeaacqWGlbWsaaaabaGaemOyai2aaSbaaSqaaiabigdaXaqabaGccqGH9aqpdaWcaaqaaiabgkHiTiabdAgaMnaaBaaaleaacqWGibasaeqaaaGcbaGaem4saSeaaaqaaiabdkgaInaaBaaaleaacqaIWaamaeqaaOGaeyypa0ZaaSaaaeaacqaIXaqmaeaacqWGlbWsaaaaaiaaxMaacaWLjaWaaeWaaeaacqaI0aanaiaawIcacaGLPaaaaaa@4928@

where K is the limb stiffness. Model parameters of equation 4 can be identified through multiple linear regression of equation 2 using recorded experimental data. In particular, insertion of the robot forces and step heights measured during a force field perturbation into equation 2 allows the coefficients a_0_, b_1_, and b_0 _to be identified using linear regression [16].

We assume now that the force field applied to the leg is the sum of two perturbations: the force applied by the assisting robot, R_i_, and a force created by the virtual impairment, I_i_:

*F*_*i *_= *R*_*i *_+ *I*_*i *_    (5)

The virtual impairment force I_i _can be imagined as the effect of a neural injury expressed as a force. For example, if an individual has difficulty lifting their leg following injury, this could be modeled as the consequence of a virtual force that pushes the leg downward, relative to the normative condition. We studied a virtual impairment that pushes the leg upward rather than downward in this paper because a downward impairment could cause tripping when the robot training device does not compensate for the field, and we wished to analyze and compare against the learning dynamics without robotic compensation.

Substituting equation 5 into equation 2 gives the dynamics of motor adaptation in response to the robot assistance and the impairment field:

*e*_*i*+1 _= *a*_0_*e*_*i *_+ *b*_1_*R*_*i *_+ *b*_0_*R*_*i*+1 _+ *b*_1_*I*_*i *_+ *b*_0_*I*_*i*+1 _    (6)

We wish to find a robot controller that minimizes the cost function in equation 1 for the dynamics in equation 6. Now, the minimum of the cost function in equation 1 occurs when:

∂J∂Ri+1=ei+1∂ei+1∂Ri+1+λRRi+1=0     (7)
 MathType@MTEF@5@5@+=feaafiart1ev1aaatCvAUfKttLearuWrP9MDH5MBPbIqV92AaeXatLxBI9gBaebbnrfifHhDYfgasaacH8akY=wiFfYdH8Gipec8Eeeu0xXdbba9frFj0=OqFfea0dXdd9vqai=hGuQ8kuc9pgc9s8qqaq=dirpe0xb9q8qiLsFr0=vr0=vr0dc8meaabaqaciaacaGaaeqabaqabeGadaaakeaadaWcaaqaaiabgkGi2kabdQeakbqaaiabgkGi2kabdkfasnaaBaaaleaacqWGPbqAcqGHRaWkcqaIXaqmaeqaaaaakiabg2da9iabdwgaLnaaBaaaleaacqWGPbqAcqGHRaWkcqaIXaqmaeqaaOWaaSaaaeaacqGHciITcqWGLbqzdaWgaaWcbaGaemyAaKMaey4kaSIaeGymaedabeaaaOqaaiabgkGi2kabdkfasnaaBaaaleaacqWGPbqAcqGHRaWkcqaIXaqmaeqaaaaakiabgUcaRiabeU7aSnaaBaaaleaacqWGsbGuaeqaaOGaemOuai1aaSbaaSqaaiabdMgaPjabgUcaRiabigdaXaqabaGccqGH9aqpcqaIWaamcaWLjaGaaCzcamaabmaabaGaeG4naCdacaGLOaGaayzkaaaaaa@5559@

Rearranging equation 7 with the partial derivative taken from equation 6 gives the robot controller that minimizes this cost function:

Ri+1=−b0λRei+1,     (8)
 MathType@MTEF@5@5@+=feaafiart1ev1aaatCvAUfKttLearuWrP9MDH5MBPbIqV92AaeXatLxBI9gBaebbnrfifHhDYfgasaacH8akY=wiFfYdH8Gipec8Eeeu0xXdbba9frFj0=OqFfea0dXdd9vqai=hGuQ8kuc9pgc9s8qqaq=dirpe0xb9q8qiLsFr0=vr0=vr0dc8meaabaqaciaacaGaaeqabaqabeGadaaakeaacqWGsbGudaWgaaWcbaGaemyAaKMaey4kaSIaeGymaedabeaakiabg2da9iabgkHiTmaalaaabaGaemOyai2aaSbaaSqaaiabicdaWaqabaaakeaacqaH7oaBdaWgaaWcbaGaemOuaifabeaaaaGccqWGLbqzdaWgaaWcbaGaemyAaKMaey4kaSIaeGymaedabeaakiabcYcaSiaaxMaacaWLjaWaaeWaaeaacqaI4aaoaiaawIcacaGLPaaaaaa@4228@

which is a simple error-feedback, discrete-time controller. At this point, the robot controller requires an estimation of the next error. Here we take advantage of our knowledge about the dynamics of motor adaptation and use the autoregressive model of equation 6 to provide an estimate of the next error to the robot. Thus, the robotic assistance implements a predictive control strategy that combines an error estimator with a controller that performs a gradient-descent optimization. This control structure is very similar to the function performed by the human during motor adaptation to a novel dynamic environment [21]. In this case, the robot controller takes the form:

*R*_*i*+1 _= *f*_*R*_*R*_*i *_- *g*_*R*_*Ke*_*i *_+ *c*_*R*_(*f*_*H*_*I*_*i *_- *I*_*i*+1_),     (9)

with the following parameters:

fR=fHλRK2+1cR=1λRK2+1gR=fH−g^HλRK2+1g^H=gHK     (10)
 MathType@MTEF@5@5@+=feaafiart1ev1aaatCvAUfKttLearuWrP9MDH5MBPbIqV92AaeXatLxBI9gBaebbnrfifHhDYfgasaacH8akY=wiFfYdH8Gipec8Eeeu0xXdbba9frFj0=OqFfea0dXdd9vqai=hGuQ8kuc9pgc9s8qqaq=dirpe0xb9q8qiLsFr0=vr0=vr0dc8meaabaqaciaacaGaaeqabaqabeGadaaakeaafaqaaeGabaaabaqbaeqabeGaaaqaaiabdAgaMnaaBaaaleaacqWGsbGuaeqaaOGaeyypa0ZaaSaaaeaacqWGMbGzdaWgaaWcbaGaemisaGeabeaaaOqaaiabeU7aSnaaBaaaleaacqWGsbGuaeqaaOGaem4saS0aaWbaaSqabeaacqaIYaGmaaGccqGHRaWkcqaIXaqmaaaabaGaem4yam2aaSbaaSqaaiabdkfasbqabaGccqGH9aqpdaWcaaqaaiabigdaXaqaaiabeU7aSnaaBaaaleaacqWGsbGuaeqaaOGaem4saS0aaWbaaSqabeaacqaIYaGmaaGccqGHRaWkcqaIXaqmaaaaaaqaauaabeqabiaaaeaacqWGNbWzdaWgaaWcbaGaemOuaifabeaakiabg2da9maalaaabaGaemOzay2aaSbaaSqaaiabdIeaibqabaGccqGHsislcuWGNbWzgaqcamaaBaaaleaacqWGibasaeqaaaGcbaGaeq4UdW2aaSbaaSqaaiabdkfasbqabaGccqWGlbWsdaahaaWcbeqaaiabikdaYaaakiabgUcaRiabigdaXaaaaeaacuWGNbWzgaqcamaaBaaaleaacqWGibasaeqaaOGaeyypa0ZaaSaaaeaacqWGNbWzdaWgaaWcbaGaemisaGeabeaaaOqaaiabdUealbaaaaaaaiaaxMaacaWLjaWaaeWaaeaacqaIXaqmcqaIWaamaiaawIcacaGLPaaaaaa@63E2@

As the robot controller minimizes a cost function similar to one identified for the human motor system [21], it is not surprising that the controller in equation 9 adjusts the robot force based on the step height error and uses a forgetting factor, f_R, _to decrement the robot force on the next movement when error is small. The control law also contains a feedforward term related to impairment force, I. This term is small if the impairment is assumed constant and the human forgetting factor is near one. One effect of this feedforward term is to initialize the robot force, R, so that it limits the initial kinematic error when the impairment is initially experienced. This is a nuance of our approach using the robotic force field paradigm as we have control over the virtual impairment. In clinical practice, the patient's impairment would already have occurred and the robot would need to be initialized, perhaps with a high impedance controller to constrain errors.

### Stability of the coupled human-robot system

With the control law of the robot and the motor adaptation dynamics established, verification of system stability in the coupled human-robot system is desired. Taking the z transform of both sides of equations 6 and 9, and imposing zero initial values for R, e, and I, we obtain:

(1 - *a*_0_*z*^-1^)*E*(*z*) = (*b*_1_*z*^-1 ^+ *b*_0_)[*R*(*z *+ *I*(*z*)]

(1 - *f*_*R*_*z*^-1^)*R*(*z*) = -*g*_*R*_*Kz*^-1 ^*E*(*z*)+ *c*_*R*_(*f*_*H*_*z*^-1 ^- 1)*I*(*z*)     (11)

where E(z), R(z) and I(z) are the z transforms of e_i+1_, R_i+1 _and I_i+1_, respectively. From the last system of equations, we obtain the two transfer functions that are relevant for the stability of the closed-loop system:

HEI(z)=E(z)I(z)=b0(1−cR)(1−fHz−1)1−(fR+a0−gR)z−1,HRI(z)=R(z)I(z)=cR(fHz−1−1)1−(fR+a0−gR)z−1.     (12)
 MathType@MTEF@5@5@+=feaafiart1ev1aaatCvAUfKttLearuWrP9MDH5MBPbIqV92AaeXatLxBI9gBaebbnrfifHhDYfgasaacH8akY=wiFfYdH8Gipec8Eeeu0xXdbba9frFj0=OqFfea0dXdd9vqai=hGuQ8kuc9pgc9s8qqaq=dirpe0xb9q8qiLsFr0=vr0=vr0dc8meaabaqaciaacaGaaeqabaqabeGadaaakeaafaqabeGabaaabaGaemisaG0aaSbaaSqaaiabdweafjabdMeajbqabaGccqGGOaakcqWG6bGEcqGGPaqkcqGH9aqpdaWcaaqaaiabdweafjabcIcaOiabdQha6jabcMcaPaqaaiabdMeajjabcIcaOiabdQha6jabcMcaPaaacqGH9aqpdaWcaaqaaiabdkgaInaaBaaaleaacqaIWaamaeqaaOGaeiikaGIaeGymaeJaeyOeI0Iaem4yam2aaSbaaSqaaiabdkfasbqabaGccqGGPaqkcqGGOaakcqaIXaqmcqGHsislcqWGMbGzdaWgaaWcbaGaemisaGeabeaakiabdQha6naaCaaaleqabaGaeyOeI0IaeGymaedaaOGaeiykaKcabaGaeGymaeJaeyOeI0IaeiikaGIaemOzay2aaSbaaSqaaiabdkfasbqabaGccqGHRaWkcqWGHbqydaWgaaWcbaGaeGimaadabeaakiabgkHiTiabdEgaNnaaBaaaleaacqWGsbGuaeqaaOGaeiykaKIaemOEaO3aaWbaaSqabeaacqGHsislcqaIXaqmaaaaaOGaeiilaWcabaGaemisaG0aaSbaaSqaaiabdkfasjabdMeajbqabaGccqGGOaakcqWG6bGEcqGGPaqkcqGH9aqpdaWcaaqaaiabdkfasjabcIcaOiabdQha6jabcMcaPaqaaiabdMeajjabcIcaOiabdQha6jabcMcaPaaacqGH9aqpdaWcaaqaaiabdogaJnaaBaaaleaacqWGsbGuaeqaaOWaaeWaaeaacqWGMbGzdaWgaaWcbaGaemisaGeabeaakiabdQha6naaCaaaleqabaGaeyOeI0IaeGymaedaaOGaeyOeI0IaeGymaedacaGLOaGaayzkaaaabaGaeGymaeJaeyOeI0IaeiikaGIaemOzay2aaSbaaSqaaiabdkfasbqabaGccqGHRaWkcqWGHbqydaWgaaWcbaGaeGimaadabeaakiabgkHiTiabdEgaNnaaBaaaleaacqWGsbGuaeqaaOGaeiykaKIaemOEaO3aaWbaaSqabeaacqGHsislcqaIXaqmaaaaaOGaeiOla4caaiaaxMaacaWLjaWaaeWaaeaacqaIXaqmcqaIYaGmaiaawIcacaGLPaaaaaa@968B@

The stability condition for the coupled feedback system requires that the poles of both transfer functions remain inside the unit circle in the z plane [24], i.e. that:

|*f*_*R *_+ *a*_0 _- *g*_*R*_| < 1.     (13)

By taking the inverse z transform of the H_EI_(z) transfer function in equation 12, the closed-loop dynamics for the subject when coupled to the robotic training system is given by:

*e*_*i*+1 _= (*f*_*R *_+ *a*_0 _- *g*_*R*_)*e*_*i *_+ *b*_0_(1 - *c*_*R*_)(*I*_*i*+1 _- *f*_*H*_*I*_*i*_)     (14)

**Figure 1:**
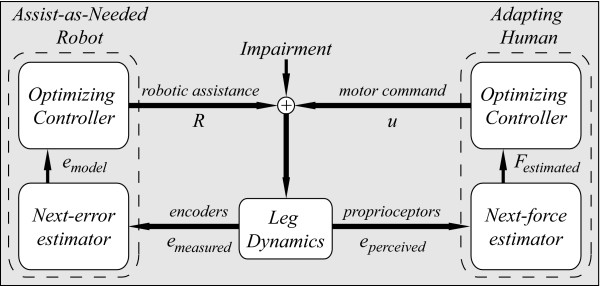
**Conceptual overview of human-robot cooperative, assist-as-needed gait training**. The goal of this type of training is to allow the human to learn to compensate for the gait impairment, but to reduce the kinematic errors experienced during this adaptation process. The addition of an assist-as-needed robot results in two adaptive interacting subsystems: the robot and the human. Each subsystem is composed of an optimizing controller and a next-step estimator. The assist-as-needed robotic controller mimics the structure of the adapting human, and it is configured such that it relaxes its assistance faster than the human learns. This allows it to challenge the human yet limit performance errors in an assist-as-needed manner.

Note that these dynamics arises from the interaction of two adaptive processes: the robot control algorithm and the human motor adaptation to the applied forces (Fig. 1).

Substituting g_R _and f_R _from equation 10 and a_0 _from equation 4 into equation 13, we obtain an expression of the stability condition in terms of the human parameters and the robot gain λ_R_:

|fH−λRK2λRK2+1g^H|<1     (15)
 MathType@MTEF@5@5@+=feaafiart1ev1aqatCvAUfKttLearuWrP9MDH5MBPbIqV92AaeXatLxBI9gBaebbnrfifHhDYfgasaacH8akY=wiFfYdH8Gipec8Eeeu0xXdbba9frFj0=OqFfea0dXdd9vqai=hGuQ8kuc9pgc9s8qqaq=dirpe0xb9q8qiLsFr0=vr0=vr0dc8meaabaqaciaacaGaaeqabaqabeGadaaakeaadaabdaqaaiabdAgaMnaaBaaaleaacqWGibasaeqaaOGaeyOeI0YaaSaaaeaacqaH7oaBdaWgaaWcbaGaemOuaifabeaakiabdUealnaaCaaaleqabaGaeGOmaidaaaGcbaGaeq4UdW2aaSbaaSqaaiabdkfasbqabaGccqWGlbWsdaahaaWcbeqaaiabikdaYaaakiabgUcaRiabigdaXaaacuWGNbWzgaqcamaaBaaaleaacqWGibasaeqaaaGccaGLhWUaayjcSdGaeyipaWJaeGymaeJaaCzcaiaaxMaadaqadaqaaiabigdaXiabiwda1aGaayjkaiaawMcaaaaa@495F@

Thus, the system is stable for all λ_R _> 0 if |f_H_-ĝ_H_| < 1 and 0 < f_H _< 1. According to equations 2, 4, and 10, the condition |f_H_-ĝ_H_| < 1 corresponds to the stability condition for the human adaptive system operating on its own (i.e. without a robot movement trainer). Thus, the situation of inappropriate robot gain selection is prevented because the optimized controller bounds the robot gains in equation 10 relative to the parameters that determine the stability of the human adaptive system. In addition, given the parameters for the human learning system g_H_, f_H _and K, the stability condition imposes a restriction on the value of the parameter λ_R _in the cost function. Specifically, λ_R _has to be chosen either:

λR>1−fHK2(a0−1),orλR<−1+fHK2(a0+1),     (16)
 MathType@MTEF@5@5@+=feaafiart1ev1aaatCvAUfKttLearuWrP9MDH5MBPbIqV92AaeXatLxBI9gBaebbnrfifHhDYfgasaacH8akY=wiFfYdH8Gipec8Eeeu0xXdbba9frFj0=OqFfea0dXdd9vqai=hGuQ8kuc9pgc9s8qqaq=dirpe0xb9q8qiLsFr0=vr0=vr0dc8meaabaqaciaacaGaaeqabaqabeGadaaakeaafaqabeqadaaabaGaeq4UdW2aaSbaaSqaaiabdkfasbqabaGccqGH+aGpdaWcaaqaaiabigdaXiabgkHiTiabdAgaMnaaBaaaleaacqWGibasaeqaaaGcbaGaem4saS0aaWbaaSqabeaacqaIYaGmaaGccqGGOaakcqWGHbqydaWgaaWcbaGaeGimaadabeaakiabgkHiTiabigdaXiabcMcaPaaacqqGSaalaeaacqqGVbWBcqqGYbGCaeaacqaH7oaBdaWgaaWcbaGaemOuaifabeaakiabgYda8iabgkHiTmaalaaabaGaeGymaeJaey4kaSIaemOzay2aaSbaaSqaaiabdIeaibqabaaakeaacqWGlbWsdaahaaWcbeqaaiabikdaYaaakiabcIcaOiabdggaHnaaBaaaleaacqaIWaamaeqaaOGaey4kaSIaeGymaeJaeiykaKcaaaaacqGGSaalcaWLjaGaaCzcamaabmaabaGaeGymaeJaeGOnaydacaGLOaGaayzkaaaaaa@58C0@

and any λ_R _outside of this range will result in an unstable controller because the pole of the transfer function has a vertical asymptote at λ_R _= -1/K^2^. As |a_0_| < 1 and f_H _< 1, the right side term in the first inequality in equation 16 is negative and therefore any λ_R _> 0 will lead to a stable controller.

### Optimality Constraints on the Control Gains

In addition to guaranteeing stability, choosing λ_R _> 0 imposes an additional relation between the human and robot forgetting factors f_H _and f_R_. This can be seen by examining equation 10 for f_R_, which was derived assuming an optimizing controller. For λ_R _> 0, we have f_R _< f_H _and therefore the robot must attempt to decrease its force more quickly than the human controller in order to assist only as needed, as we found previously in simulation [25]. In other words, this relation can be understood as the requirement that the robotic trainer must decrease its assistance (equation 9) faster than the human decreases its muscle force (equation 3). Thus, the robot must adapt faster than the human motor system in order to continually challenge it to learn.

Although the coupled system may be stable for negative choices of λ_R _within the region defined by inequality of equation 16, this will result in a situation where f_R _> f_H_. Thus, such choices will lead to a situation in which the coupled system is stable but the assistance as needed algorithm will not allow the subject to learn to compensate for the virtual impairment.

### Robotic assistance with a continuous error weighting function

As described in the previous sections, the robot assistance depends from step to step on the value of the subject's performance error according to equation 9. A problem of the previous strategy is that the amount of the virtual impairment compensated by the robot is always non-zero. Indeed, as the robot controller minimizes error and force, the steady state condition presents a finite amount of robotic assistance. This would have not happened if the robot only attempted to minimize force, as steady state assistance would always be zero. In addition, subjects present a characteristic variability in their step heights that needs to be considered by the controller. Thus, both the form of the robotic controller and the subject's error variability contribute to steady state values of robotic assistance greater than zero.

**Figure 2:**
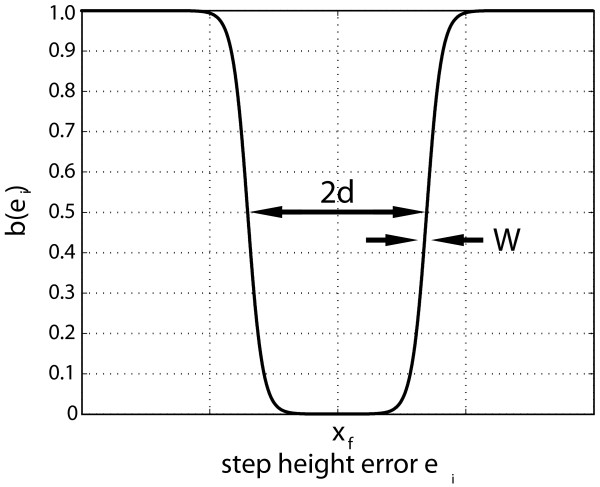
**Error weighting function**. We designed a weighting function, β(e_i_), which scales the feedback error contribution to the robotic assistance force. If the step height error falls within the 2δ (6σ) window then the robot assistance decays as a function of equation 18. The transition zone defined by W = 1/2 σ in equation 18, allowed a smooth transition from 0 to 1 using a weighted hyperbolic tangent function.

In order to allow the subject to learn the complete virtual impairment and to allow the robotic assistance to fade to zero, we introduce a new next-force estimator in the evaluation of the feedback law of equation 6, based on the assumption that error variability is acceptable within a known band. The new next-error estimator is given by:

*e*_*i*+1 _= β(*e*_*i*_)(*a*_0_*e*_*i *_+ *b*_1_*I*_*i *_+ *b*_0_*I*_*i*+1_) + *b*_1_*R*_*i *_+ *b*_0_*R*_*i*+1_,     (17)

where β(*e*_*i*_) is a non-linear function that smoothly changes from 0 to 1 as the absolute value of the performance error increases (Fig. 2). The weighting function is given by:

β(ei)=1+12[tanh⁡(W(ei−δ))−tanh⁡(W(ei+δ))]     (18)
 MathType@MTEF@5@5@+=feaafiart1ev1aaatCvAUfKttLearuWrP9MDH5MBPbIqV92AaeXatLxBI9gBaebbnrfifHhDYfgasaacH8akY=wiFfYdH8Gipec8Eeeu0xXdbba9frFj0=OqFfea0dXdd9vqai=hGuQ8kuc9pgc9s8qqaq=dirpe0xb9q8qiLsFr0=vr0=vr0dc8meaabaqaciaacaGaaeqabaqabeGadaaakeaacqaHYoGycqGGOaakcqWGLbqzdaWgaaWcbaGaemyAaKgabeaakiabcMcaPiabg2da9iabigdaXiabgUcaRmaalaaabaGaeGymaedabaGaeGOmaidaamaadmaabaGagiiDaqNaeiyyaeMaeiOBa4MaeiiAaGMaeiikaGIaem4vaCLaeiikaGIaemyzau2aaSbaaSqaaiabdMgaPbqabaGccqGHsislcqaH0oazcqGGPaqkcqGGPaqkcqGHsislcyGG0baDcqGGHbqycqGGUbGBcqGGObaAcqGGOaakcqWGxbWvcqGGOaakcqWGLbqzdaWgaaWcbaGaemyAaKgabeaakiabgUcaRiabes7aKjabcMcaPiabcMcaPaGaay5waiaaw2faaiaaxMaacaWLjaWaaeWaaeaacqaIXaqmcqaI4aaoaiaawIcacaGLPaaaaaa@5E4E@

where δ defines the width of an acceptable error band and *W *characterizes the smoothness of the transition region from 0 and 1. Thus, the function defines an acceptable error band of width, 2δ, in which the weighting function decays to zero.

Every individual has characteristic fluctuations in their step heights, which are assumed to be normally distributed with mean x_f _and standard deviation σ. These inherent fluctuations in step height need to be considered when training a given force field. That is, step height errors within and around the mean performance window of step heights should not be penalized, as noise around a mean is considered natural. Choosing δ to be equal to 3σ, the band takes into account 99.7% of the typical step height fluctuations.

Inserting the estimator from equation 17 into the optimal robot controller in equation 9, we derive a controller that adapts its output as a function of performance error with respect to a desired mean with normally distributed error fluctuations:

*R*_*i*+1 _= *f*_*R*_*R*_*i *_- β(*e*_*i*_)(*g*_*R*_*Ke*_*i *_- *c*_*R*_(*f*_*H*_*I*_*i *_- *I*_*i*+1_)).     (19)

The resulting action of the controller can be described as follows. When an individual's error is outside the band of typical variability, the estimator predicts the next error by using the ARX model equation 17 with β(e_i_) = 1 and therefore applies the previous defined robot controller of equation 9. If the individual's error is within the band, we weight the error as a function of equations 17 and 18. When errors are both within the band and small, β(e_i_) approaches zero and the robot gradually decreases its assistance following the recursive law:

*R*_*i*+1 _= *f*_*R*_*R*_*i *_    (20)

thus fading the assistance completely to zero and allowing the individual to experience the entirety of the impairment force field.

The weighted error band introduces a non-linearity into the feedback path of the robotic controller. The absolute stability of the resulting non-linear control system can be studied using standard approaches such as the Circle's or Popov's criteria [24]. These methods can be applied to non-linearities that are bounded between two straight lines passing through the origin with slopes a and b that satisfy a < b. In our case, the form of the non-linearity in the feedback path is given by β(e_i_)e_i, _and therefore it is always bounded between two lines with slopes a = 0 and b = 1. We have tested the stability of the non-linear controller for different values of the control gains, confirming that the system is stable as long as the stability of linear system is ensured.

### Experimental Setup

**Figure 3:**
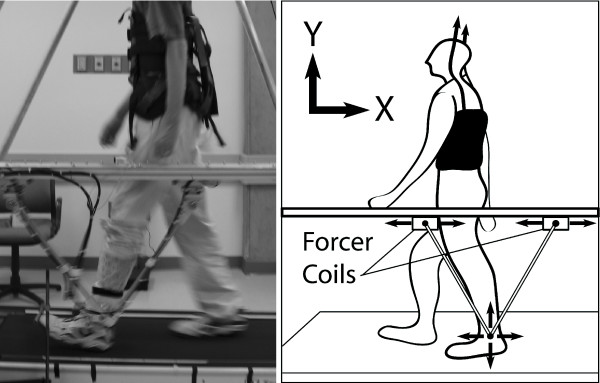
**Experimental Apparatus for Creating a Virtual Impairment and for Assisting in Motor Learning**. Picture (left) and diagram (right) of experimental setup. The robot makes use of a linear motor with two forcer coils and a V-shaped linkage to accommodate and drive motion of the robot's apex in the parasagittal plane. The apex is attached through a padded brace and revolute joint to the subject's lower shank. Subjects wore a harness attached to an overhead frame to catch them in case they fell. The robot created a virtual impairment by pushing upward on the subject's lower shank during the swing phase of gait, making the subject step abnormally high. The robot also assisted the subject in learning to compensate for the virtual impairment with small step height errors by helping counteract the impairment.

We tested the above theoretical results with an extensive set of experiments. Ten healthy, unimpaired subjects (7 male, 23–39 yrs) completed three experiments during which a lightweight, two degrees-of-freedom, planar robot (Fig. 3) applied an upwardly directed, viscous force field (i.e. the virtual impairment) to the subject's lower shank during walking on a treadmill (Table 1). Details of this device can be found in [26]. Subjects were instructed to walk as consistently as possible without directly looking at their feet. Subjects wore a harness suspended from an overhead body weight support system that served to catch them if they fell. However, the amount of body weight support was set to zero such that the harness did not impede normal stepping. The University of California – Irvine IRB approved all experiments and the subjects provided informed consent.

**Table 1:** Experimental Protocol

**Experiment**	**# of Subjects**	**Protocol**
Preliminary	10	100N-100I-100N, I peak was approximately 6% of subject's body weight
A: AAN	10	190N(9CTs)-100I-100N-200I+R(9CTs)-50N, I peak was 6% of subject's body weight
B: AAN with Noise Insensitivity	10	Experiment A with a 6σ based weighted error band
C: AAN with Improper Parameter Selection	4	Experiment A with f_R _= 0.90

AAN = Assist-as-Needed, CTs = Catch Trials, N = Null Field (no force field applied), I = Upwardly directed Impairment field, R = Downwardly directed compensating Robotic field

The force field that created the virtual impairment was proportional to the subject's forward velocity during the swing phase of gait, such that the force in the vertical direction was:

I={−BIx˙,x˙<00,x˙>0     (21)
 MathType@MTEF@5@5@+=feaafiart1ev1aaatCvAUfKttLearuWrP9MDH5MBPbIqV92AaeXatLxBI9gBaebbnrfifHhDYfgasaacH8akY=wiFfYdH8Gipec8Eeeu0xXdbba9frFj0=OqFfea0dXdd9vqai=hGuQ8kuc9pgc9s8qqaq=dirpe0xb9q8qiLsFr0=vr0=vr0dc8meaabaqaciaacaGaaeqabaqabeGadaaakeaacqWGjbqscqGH9aqpdaGabeqaauaabeqaciaaaeaacqGHsislcqWGcbGqdaWgaaWcbaGaemysaKeabeaakiqbdIha4zaacaGaeiilaWcabaGafmiEaGNbaiaacqGH8aapcqaIWaamaeaacqaIWaamcqGGSaalaeaacuWG4baEgaGaaiabg6da+iabicdaWaaaaiaawUhaaiaaxMaacaWLjaWaaeWaaeaacqaIYaGmcqaIXaqmaiaawIcacaGLPaaaaaa@430E@

The B_I _gain was chosen such that the peak force exerted on the subject during swing was equivalent to approximately 6% of the subject's body weight. We have found this magnitude to induce measurable perturbations to the stepping trajectory but not cause stumbling [16]. The effect of the virtual impairment was to push the leg higher than normal during swing.

The robotic assistance for stepping was applied as a second force field of the same form but opposite sign:

R={BRx˙,x˙<00,x˙>0     (22)
 MathType@MTEF@5@5@+=feaafiart1ev1aaatCvAUfKttLearuWrP9MDH5MBPbIqV92AaeXatLxBI9gBaebbnrfifHhDYfgasaacH8akY=wiFfYdH8Gipec8Eeeu0xXdbba9frFj0=OqFfea0dXdd9vqai=hGuQ8kuc9pgc9s8qqaq=dirpe0xb9q8qiLsFr0=vr0=vr0dc8meaabaqaciaacaGaaeqabaqabeGadaaakeaacqWGsbGucqGH9aqpdaGabeqaauaabeqaciaaaeaacqWGcbGqdaWgaaWcbaGaemOuaifabeaakiqbdIha4zaacaGaeiilaWcabaGafmiEaGNbaiaacqGH8aapcqaIWaamaeaacqaIWaamcqGGSaalaeaacuWG4baEgaGaaiabg6da+iabicdaWaaaaiaawUhaaiaaxMaacaWLjaWaaeWaaeaacqaIYaGmcqaIYaGmaiaawIcacaGLPaaaaaa@4247@

where the B_R _gain determined the strength of the assistance. Thus the net force field applied to the subject had strength of B_R_-B_I_. The virtual impairment pushed upward and the robotic assistance helped by pulling downward during the swing phase of gait. At a constant treadmill and thus walking speed, forces and gains are directly proportional. Thus the B_R _and B_I _gains were mapped directly to the I and R forces in equation 6.

Selection of the parameters for the assist-as-needed control algorithm developed in the previous section requires knowledge of the K, f_H_, and g_H _parameters that describe normal human adaptation. We previously analysed motor adaptation by unimpaired subjects to perpendicular viscous force fields applied to the leg during treadmill walking [16,21]. Here, we used the mean parameters from ten subjects adapting to a 6% body weight viscous field (i.e. the same field type and strength used in this study). The mean parameters across subjects were K = 3.0 N/cm +/- 0.62 SD, g_H _= 0.80 N/cm +/- 0.80 SD, and f_H _= 0.76 +/- 0.21 SD. We chose the value of λ_R _to be 0.1, a magnitude that in simulation allowed a reasonable balance between error and assistance force. Smaller values caused the simulated assistance-as-needed controller to respond very strongly to kinematic errors, never reducing its assistance, while larger values caused it to allow large kinematic errors. This positive chosen value for λ_R _allows f_R _< f_H _and the coupled system to be stable per equation 16.

### Experimental Protocol

In a preliminary experiment (Table 1), subjects first walked for 100 steps in a null field (N), for which no field (B_I _and B_R _= 0) was applied, to become comfortable walking in the robot. We have shown previously that the robot alters the normal pattern of stepping only slightly in this null field condition, because the robot has low inertia and we apply simple friction and gravity compensation in software during this null field condition [26]. Subjects were then exposed to a force field (F) for 100 steps. A gain value for the impairment field, B_I_, was chosen that approximated 6% body weight. Finally, the subjects returned the null field for 100 steps. The data from this experiment was used to identify the mean peak swing velocity for each subject, in order to calculate the impairment field gain that corresponded precisely to 6% of the subjects' body weight for the subsequent experiments. As a result of this calibration process, the mean virtual impairment field strength applied across subjects was 6.2% +/- 0.29 SD of body weight with the field gain B_I _ranging from 32 to 46 N-s/m.

Next, an experiment was performed to compare how the subjects adapted to the force field with and without robotic assistance. For this first assist-as-needed (AAN) experiment, subjects first walked for 190 steps in a null field stage. The robot applied 10 randomly spaced "catch trials" during which the field was turned on for a single step. This allowed measurement of the 'direct effect' or the step height on initial field exposure. During the second stage, the robot applied a constant gain viscous force field with strength proportional to 6% of the subjects' body weight for 100 steps (i.e. the virtual impairment). This stage allowed quantification of how well subjects could learn to compensate for the virtual impairment without robot assistance. From this stage, we also identified the standard deviation of the step height error in the presence of the force field. This stage was then followed by a null field stage for 100 steps to wash out any learning of the virtual impairment. We [16] and others [27] have shown that prolonged exposure to a null field following adaptation effectively "resets" the motor control system so that it adapts as if from a novice state when exposed to a force field again.

We then applied the virtual impairment with robotic assistance. During this fourth stage, the virtual impairment was again turned on for 200 steps, and robot assistance was provided as a function of the subject's step height error. Specifically, the robot provided a superimposed, assisting force field using the "assist-as-needed" robot control law in equation 9. The robot control law determined the gain of this assistance force field, which was of the same form (i.e. vertical viscous force) as the perturbing impairment force field. Following the initial 100 steps, ten randomly spaced "catch trials" were used to test for the presence of after effects and thus internal model formation of the virtual impairment. Finally, the subject walked for 50 steps in a null field stage to again reset the motor system to a novice state.

Two modifications of this protocol were performed. The first modification, which we will term the "Assistance-as-Needed (AAN) with Noise Insensitivity" protocol, studied whether the modified assist-as-needed control law in equation 19 more effectively handled the inherent variability in the subject's stepping. Specifically, the weighting function in equation 18 was applied to the error feedback and feedforward terms of the robot control law per equation 19. This, in essence, created two styles of robotic assistance that were dependent on subject performance error. If step height error was within a band whose mean was defined by the subject's mean step height in the field and whose width was determined by the subject's stepping variability, the robot assistance decayed quickly to zero allowing the subject to experience the full virtual impairment. However, if the error was near the edge or outside of this band, the robot assistance still attempted to decay but was responsive to the magnitude of the error with respect to the center of the band. This continuous alteration of the robotic assistance was designed to reduce or eliminate assistance as the subject began to perform along a desired movement trajectory with typical fluctuations in that performance.

Only four of the ten subjects participated in the second modification, which we will term the "Assistance-as-Needed with Improper Parameter Selection" protocol. This protocol tested the prediction that the value of the robot forgetting factor f_R _cannot be chosen arbitrarily and indeed is required to be less than the human forgetting factor f_H _if the assistance-as-needed technique is to function properly. A value of f_R _= 0.90 was chosen to be greater than the mean f_H _that we have identified in previous experiments, but one that still maintains the stability of the coupled system. Therefore, this value of f_R _allowed us to test, as predicted by the equations, that the robot would 'take over' and not allow the subject to experience the virtual impairment.

In all cases, the step height error used by the robot to prescribe the assistance-as-needed was defined as the relative difference in step height on each step to the mean steady state step height, x_f_, achieved following adaptation to the virtual impairment. This x_f _was calculated for each subject for each experiment by taking a running average of steps 50–90 for the stage in which the virtual impairment was applied without robot assistance. For the Noise-Insensitive AAN experiment only, the standard deviation of step height error was calculated from the last half of the steps during exposure to the virtual impairment without robot assistance in the first AAN experiment (Table 1). This calculation assumed that subject variability would not significantly change from one experiment to the next.

### Data and Statistical Analysis

The position of the robot attachment and magnitude of force applied by the robot were collected at 200 Hz. Position and the calculated velocity were processed into steps based on zero crossings of horizontal velocity. Steps were parameterised by taking step height and force measures at 300 ms and 100 ms, respectively, following forward movement of the robot's apex at the beginning of swing. Step height error was calculated with respect to the mean of the null field step heights in the first exposure to the null field; thus the mean step height error in the first null field was zero. Abnormally high steps were defined as having positive error, and low steps as having a negative error.

To compare the difference in step height errors across conditions with and without robotic assistance, the following comparisons were performed. First to compare if initial step height errors were lower with robotic assistance, the ten null field direct effect errors were averaged per subject. A one-way analysis of variance (ANOVA) then compared the mean null field direct effect height to the direct effect height with robotic assistance across subjects. A similar ANOVA across subjects was performed on the individual and mean after effect heights following robotic assistance and the after effect following exposure to the full impairment field. After effects were defined as the difference between the step height error on the first trial the field was unexpectedly turned off following adaptation, and the mean of the last twenty five trials in the null field before adaptation. These comparisons were made for all three experiments in Table 1. Comparisons of robotic assistance magnitude with respect to zero were made using a one-sided t-test with the mean of the last half of the robotic assistance forces (steps 50–100).

## Results

### Robotic Assistance-as-Needed

We created a virtual impairment for ten unimpaired subjects by pushing upward on their legs during the swing phase of gait with a robotic device as they walked on a treadmill. The virtual impairment caused them to step abnormally high. We then compared how the subjects adapted to the virtual impairment with and without the robotic assistance-as-needed controller (equation 9) that we derived using an optimization approach.

**Figure 4:**
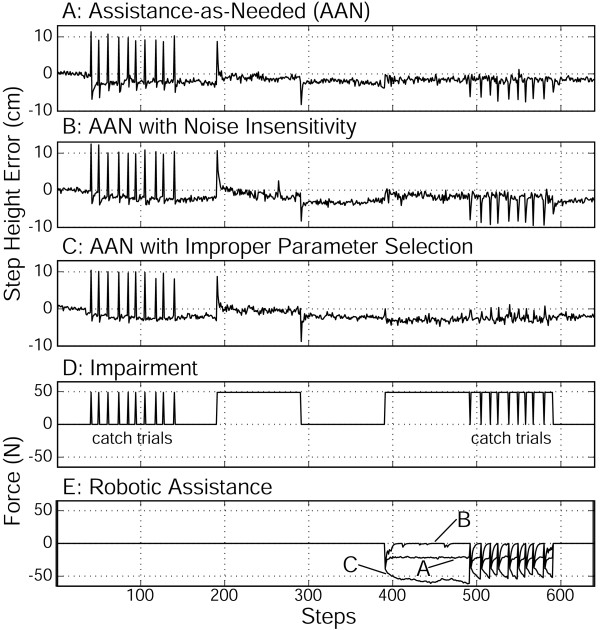
**Experimental protocol and results for a representative subject**. This subject (30 year old male) participated in each of the three experimental protocols – A: Assistance-as-needed (AAN), B: AAN with Noise Insensitivity, and C: AAN with Improper Parameter Selection. For each protocol, the subject first adapted to the virtual impairment without assistance (steps 191–290). The after effect of adaptation, indicative of internal model formation, is apparent at step 291 in A, B, and C. Then, following a period in which adaptation was washed out (steps 291–390), the subject adapted again to the virtual impairment (steps 391–591), but with the form of robotic assistance associated with the particular protocol. In all three protocols, the step height errors when the virtual impairment was turned on were much smaller (step 391, A, B, C). The robot assistance decreased for the basic AAN algorithm, but not all the way to zero (E, trace labelled "A"). The robot assistance decreased to zero for the AAN with Noise Insensitivity algorithm (E, trace labelled "B"). The robot took over the task for the AAN with Improper Parameter Selection algorithm (i.e. with f_R _> f_H_) (E, trace labelled "C"). Note that the virtual impairment force (D) was unexpectedly turned on or off ("catch trials") to measure direct effects (steps 40–140) and after effects (steps 491–591).

Without robot assistance, the subjects experienced large step height errors, but gradually learned to compensate for the virtual impairment by forming an internal model of it. Specifically, when the subjects first experienced the virtual impairment without robotic assistance, they stepped abnormally high (the "direct effect", step 191), but gradually returned their step height toward normal with repeated stepping (Fig. 4A, steps 191–240). When the virtual impairment was unexpectedly removed, the subjects exhibited after effects of adaptation (Fig. 4A, step 291). The initial after effect indicated that the subjects learned to predict the force to compensate for the virtual impairment. The subsequent after effects decreased to zero with repeated stepping in the null field.

**Figure 5:**
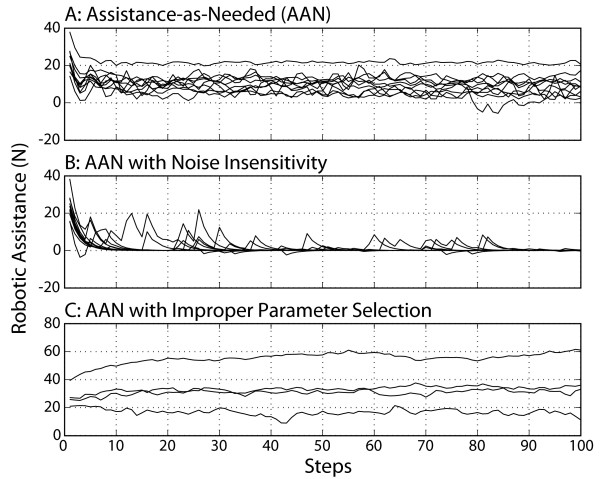
**The first hundred steps with robotic assistance**. The steps shown correspond to steps 391–491 in Figure 4. The values from Figure 4E are made negative to better visualize the decay of robotic assistance. A: Assistance as Needed protocol. Each line is data from one of the ten subjects. B: Assistance as Needed with Noise Insensitivity protocol. This assistance algorithm took into account variability in stepping height around the desired mean. Here we used a weighed error band and decreased the importance of errors within a range of normal fluctuation. When subjects exited this range, the robot assistance transiently increased C: Assistance-as-needed with Improper Parameter Selection protocol. We set the robot forgetting factor greater than the human forgetting factor (f_R _> f_H_). This created a situation in which the robot took over the task of compensating for the virtual impairment from the subjects, not allowing the subjects to learn a model of the virtual impairment. Four subjects participated in this last protocol.

**Figure 6:**
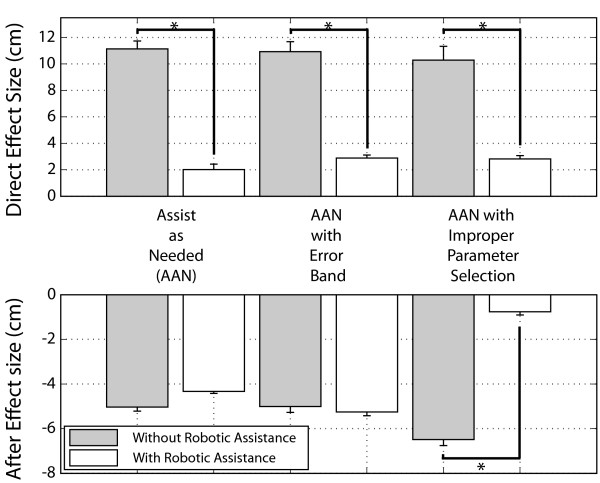
**Direct effect and after effects**. Comparison of direct effect and after effect sizes as a function of robotic assistance. Asterisk (*) indicates a significant difference, p < 0.05, as measured by a one-way ANOVA between samples. The first two AAN experiments had ten subjects. The third experiment had four subjects. Standard error bars are shown for all means.

We repeated the exposure to the virtual impairment, but with the robotic assistance turned on. With robot assistance, the subjects experienced only small errors, and gradually learned to compensate for the virtual impairment (Fig. 4A, steps 391–440). The robotic assistance decreased with repeated stepping (Fig. 5A). The subject's initial step height errors were significantly smaller with robotic assistance (Fig. 6-top, p < 0.001, ANOVA). When the virtual impairment and robotic assistance were unexpectedly turned off, the subjects again exhibited after effects of adaptation (Fig. 4, step 492).

The subjects' compensation for the virtual impairment was only partial when robotic assistance was provided, however, because the robot assistance force never decreased fully to zero. Specifically, the mean of the last half of the robotic assistance in the first experiment before the measurement of catch trials, was significantly greater than zero (Figs. 4E &5A, p < 0.001, t-test). The non-zero, steady state of robotic assistance was due to the fact that the robot attempted to minimize error as well as its own force, and the variability of the subject's performance errors around the mean. Due to the non-zero robotic assistance, subjects were never allowed to experience the full magnitude of the virtual impairment. As a consequence, there was a non-significant trend for the after effect measured after exposure to the virtual impairment to be less than those measured after adaptation without assistance (p = 0.30, ANOVA, Fig. 6-bottom).

### Assistance-as-Needed with Noise Insensitivity

Since the robot assistance did not go to zero, we modified the assist-as-needed controller so that it ignored step height errors that fell within the normal band of variability. Specifically, a non-linear weighting function was introduced to scale the importance of errors based on their size. On a per subject basis, the standard deviation of step height errors following adaptation during the first exposure to the virtual impairment was used to determine the width of the error band (δ = 3σ) and the width of the transition region (W = 1/2 σ). Across subjects, the standard deviation of step height errors following adaptation during the first exposure to the virtual impairment was σ = 1.3 +/- 0.46 cm.

Incorporation of this error weighting function caused the robotic assistance to decay to zero for all subjects (Fig. 5). As might be expected, however, the subjects exhibited step height errors near the edges of the error band. In these cases, the algorithm transiently increased its assistance to "assist" in pushing the subject back to the center of the desired band of step height kinematics. These transients are shown for all subjects in Fig. 5. These transients were particular to each subject's performance error and exemplify the concept of assist-as-needed.

Allowing robotic assistance to decay to zero allowed the subjects to experience the entirety of the virtual impairment. Thus, as would be expected, after effect magnitudes were not significantly different as compared to after effect magnitudes following adaptation to the impairment field only (Fig. 6-bottom, p > 0.05, ANOVA). That is, the subjects fully learned to counteract the virtual impairment by forming an internal model of it only when they were able to experience the impairment field in its entirety.

### Assistance-as-Needed with Improper Parameter Selection: Is f_R _> f_H _a requirement?

Four subjects performed a third experiment in which the forgetting factor of the robot was set to 0.90, a value that was less than one to ensure controller stability, but greater than the average human forgetting factor value of 0.76 which we had identified previously [16]. In this case, the assist-as-needed controller still attempted to reduce its assistance force when the step height errors were small, but at a rate slower than that of the average unimpaired learning human. Equation 9 predicts that the robot will "take over" for the subject with this parameter selection, fully compensating for the virtual impairment, and thus not allowing the subject to experience the impairment field. For the four subjects, the robot assistance cancelled 62%, 88%, 99%, and 117% of the virtual impairment following adaptation, after the robot-human system had reached a steady state (Fig. 4). Thus the robot did not allow the subjects to learn to compensate for the virtual impairment. For all subjects, this robotic over-compensation for the virtual impairment caused a loss of after effects upon field removal (Fig. 4C, trials 492–592). The after effect magnitudes were significantly less than the after effects experienced following full exposure to the impairment field (Fig. 6-bottom, p < 0.05, ANOVA).

## Discussion

The main contribution of this paper is the design of a human-robot cooperative motor training algorithm from a mathematical framework based on computational neuroscience. Specifically, we showed how an experimentally identified, mathematical model of motor adaptation can be used to derive an optimal strategy for robotic assistance, for the case of learning a novel sensory motor transformation during walking. We acknowledge that this approach is optimal in a technical sense but not necessarily in a therapeutic or motor learning sense, as certain parameters of the model remain heuristic – specifically, the selection of the cost function itself and the constant λ_R _that weights the relative cost of robot force and step height error. Solving the assist-as-needed problem using an optimization approach leads to an error-based robotic controller with a forgetting factor. This controller minimizes a weighted sum of kinematic error and robotic assistance, thus constraining error but reducing assistance when errors are small. Secondary contributions of this paper are to show how an error-weighting function can make the assist-as-needed algorithm insensitive to the normal performance variability in human stepping, and to show that the parameters of the robotic controller must be appropriately chosen so that the robot does not "take-over" the task for the human.

### Systematic reduction in assistance force

The robot controller developed here essentially allows the subject to slowly experience the virtual impairment by gradually cancelling less of it. By gradually exposing the subject to the virtual impairment, kinematic errors remain small. The action of the controller is similar to full manual assistance provided by a physical trainer early in the rehabilitation process, followed by a gradual relaxation of that assistance as the patients regains movement ability. Eventually, assistance is only provided when the patient makes movements that are substantially different from the desired movements.

The results of this study should be compared to those of a recent study that showed that unimpaired subjects could form an internal model of a force field applied to the arm during reaching without experiencing large errors through a slow ramping up of the applied force field [28]. The approach developed here is different than a slow ramping up of the force field, however, because the assist-as-needed controller is reactive to performance errors, increasing or decreasing its assistance when needed.

The controller developed here should also be compared to previous attempts to make robot-assisted therapy adaptive. Krebs et al. (2003) adapted the movement time imposed by the MIT-MANUS robot for reaching tasks based on measures of patient performance, terming this strategy "performance-based progressive robotic therapy" [18]. Kahn et al. (2004) adapted the level of force assistance provided by the ARM Guide device during reaching exercise with an error-based learning law in which the error was the difference between the actual reaching velocity and a desired, normative reaching velocity [29]. Jezernik et al. adapted the kinematic pattern of the enforced step based on measurements of the patient's interaction forces with the Lokomat [19]. The main conceptual advantage of the current approach compared to these previous approaches is that the controller developed here is based on a computational model of motor adaptation, i.e. it is based on a validated model of how the nervous system actually adapts, and is thus guaranteed optimal in a definable sense, although as we noted above, selection of the relative costs of force and error still remains heuristic.

A possible negative consequence of assist-as-needed is that motor adaptation to the virtual impairment occurs more slowly than without assistance, since subjects are only gradually exposed to the impairment. We have shown previously that motor adaptation to the force field studied here can be accelerated by transiently amplifying step height errors [16]. Thus, for some tasks, assistance-as-needed may be inappropriate. Specifically, there are some movement tasks in which large kinematic errors can be tolerated without a substantial safety or motivational risk to the subject. For these tasks, amplifying errors may accelerate motor learning, rather than reducing them with assistance as needed [7,16].

The derivation of the assistance-as-needed controller ignored the fact that human movement is inherently variable. As a result, the controller responded to the normal performance variability of stepping, and never fully decreased its assistance to zero. To solve this problem, we included an error weighting function that decreased the importance of errors within a range of acceptable variability. As a result, the robotic assistance declined to zero during training with the virtual impairment. Other weighting functions or approaches that use robust or stochastic optimal control theory might also be used to address the issue of performance noise.

We chose in this study to base the parameters of the robotic assist-as-needed controller on the mean adaptation parameters that we identified previously for ten subjects using the same type of force field perturbation [16]. This approach was sufficient to achieve a reasonable pattern of assistance-as-needed for all the subjects in the present study. It is also feasible to instead identify the model parameters on a subject-specific basis, which may allow the assistance to be more precisely tailored.

### The importance of challenge in learning

As predicted previously in simulations [25] we show above that λ > 0 leads to a stable controller and that itself leads to f_R _< f_H_. Here we provide experimental evidence that adaptive robotic assistance only works when the robot decreases its assistance faster than the rate at which the subject decreases force in the absence of error. In the model system presented here, this corresponds to f_R _< f_H_. Thus the robot must 'out forget' the forgetful human who is trying to reduce their level of effort on each movement attempt. If this condition is not held, the results here show that the robotic trainer over controls the human error by continuing to assist against the impairment. This creates a condition in which the human comes to completely rely on the robotic assistance and is not motivated to learn.

An assist-as-needed controller with f_R _> f_H _might be compared to a human trainer that always assists. For task-specific training following spinal cord injury, it has been hypothesized that rigid assistance might steer the spinal cord into a state of "learned helplessness" [30-32] in which the nervous system, not challenged to perform on its own, defers its effort to the trainer and ceases to learn. The ability to produce and be aware of one's errors and the effect of this awareness on the voluntary participation of subjects has been shown to be important in motor learning in unimpaired subjects [33-35]. As evidenced here, one method to stimulate involvement and learning is to assist-as-needed by having the robot challenge the human by allowing the human to experience some level of performance error.

### Extrapolation to the clinic

We approached the assist-as-needed principle by assuming that the process of motor recovery following neurologic injury is akin to the process of learning a sensory motor transformation. We defined a motor learning task that was amenable to computational analysis – unimpaired subjects had to overcome a virtual impairment applied to their leg with a robot. Defining a simplified task to study allowed us to rigorously develop a robotic training algorithm that mimics the assistance-as-needed provided by rehabilitation trainers. How might this strategy be extended to actual rehabilitation?

One requirement for extending this work to rehabilitation is to develop quantitative models of motor learning following neurologic injury. In order to find the robot controller that minimizes a cost function of kinematic error and robot force during rehabilitation, the dynamics of motor adaptation during rehabilitation must be defined. A key question is whether motor rehabilitation is indeed akin to learning a new sensory motor transformation, as we assumed here. In addition, the learning of the new sensory motor transformation studied here occurs after only a few steps in the virtual impairment, while rehabilitation takes months. If the index i in the model of adaptation provided by equation 9 is made to represent training session number rather than step number, can this equation be used to model motor rehabilitation? Another issue is that neurologic injury produces a range of sensory motor impairments that are difficult to model, including weakness, abnormal synergies, impaired proprioception, fatigue, spasticity, reflex hyperexcitability, hypertonia, and contracture. Development of quantitative mathematical models of motor learning in the presence of these impairments is essential for finding the robot controllers that can best assist in rehabilitation following stroke and spinal cord injury. We speculate that the form of the assist-as-needed controller derived here – an error-based learning law with a forgetting factor – will be generalizable to rehabilitation tasks. It arises from what are the simplest possible, non-trivial performance dynamics, and the simplest possible optimization, and thus may have some fundamental utility.

We have already applied this control law to modify the impedance of the ARTHuR robot as it assisted in driving the limbs of people with a spinal cord injury along a pre-recorded trajectory while they walked on a treadmill. We found that the controller reliable shaped the impedance of the robot so that the subjects stepped with normal kinematics, but with the robot's impedance large only in problematic areas of the stepping workspace (unpublished results).

## Conclusion

Here we developed and experimentally validated a robotic training algorithm that assists-as-needed in training unimpaired subjects to compensate for a virtual impairment during walking on a treadmill. The derivation of this controller stems from an optimization approach, which attempts to minimize a weighted sum of kinematic error and robotic assistance. The inclusion of an error weighting function allowed robotic assistance to fade to nothing and to allow normal stepping variability. The importance of selecting the robot parameters such that the robot relaxed its assistance faster than the subject and thus continuously challenged the subject was confirmed. Extending this approach to clinical rehabilitation will require the development of mathematical models of motor learning following stroke and spinal cord injury.

## Competing interests

The author(s) declare that they have no competing interests.

## Authors' contributions

JLE developed the study design, performed data acquisition, completed the data analysis, and wrote the manuscript. RB aided in the study design, and in the development of the β weighting function as well as in drafting and revising the manuscript. DJR conceived the optimization approach and aided in the study design and revising the manuscript.

## References

[B1] Krebs HI, Hogan N, Aisen ML, Volpe BT (1998). Robot-aided neurorehabilitation. IEEE Trans Rehabil Eng.

[B2] Patton JL, Mussa-Ivaldi FA, Rymer WZ (2001). Altering movement patterns in healthy and brain-injured subjects via custom designed robotic forces: Oct; Istanbul, Turkey..

[B3] Reinkensmeyer DJ, Aoyagi D, Emken JL, Galvez J, Ichinose WE, Kerdanyan G, Nessler JA, Maneekobkunwong S, Timoszyk W, Vallance K, Weber R, Wynne JH, de Leon RD, Bobrow JE, Harkema S, Edgerton VR (2004). Robotic Gait Training: Toward More Natural Movements and Optimal Training Algorithms: Sept; San Francisco, CA..

[B4] Reinkensmeyer DJ, Emken JL, Cramer SC (2004). Robotics, Motor Learning, and Neurologic Recovery. Ann Rev Biomed Engr.

[B5] Trombly CA (1995). Occupational therapy for dysfunction, 4th Edition.

[B6] Riener R, Lunenburger L, Jezernik S, Anderschitz M, Colombo G, Dietz V (2005). Patient-cooperative strategies for robot-aided treadmill training: first experimental results.. IEEE Trans Neural Systems & Rehab Engng.

[B7] Patton JL, Stoykov ME, Kovic M, Mussa-Ivaldi FA (2006). Evaluation of robotic training forces that either enhance or reduce error in chronic hemiparetic stroke survivors.. Exp Brain Res.

[B8] Kahn LE, Lum PS, Reinkensmeyer DJ (2003). Selection of Robotic Therapy Algorithms for the Upper Extremity in Chronic Stroke: Insights from MIME and ARM Guide Results: ; Kaist, Daejeon, Republic of Korea..

[B9] Hesse S, Werner C, Uhlenbrock D, von Frankenberg S, Bardeleben A, Brandl-Hesse B (2001). An electromechanical gait trainer for restoration of gait in hemiparetic stroke patients: preliminary results. Neurorehabilitation and Neural Repair.

[B10] Lum PS, Burgar CG, Shor PC (2004). Evidence for improved muscle activation patterns after retraining of reaching movements with the MIME robotic system in subjects with post-stroke hemiparesis. IEEE Trans Neural Systems & Rehab Engng.

[B11] Colombo G, Wirz M, Dietz V (2001). Driven gait orthosis for improvement of locomotor training in paraplegic patients. Spinal Cord.

[B12] Amirabdollahian F, Loureiro R, Driessen B, Harwin W, Mokhtari M (2001). Error correction movement for machine assisted stroke rehabilitation.. Integration of Assistive Technology in the Information Age.

[B13] Colombo R, Pisano F, Micera S, Mazzone A, Delconte C, Carrozza MC, Dario P, Minuco G (2005). Robotic techniques for upper limb evaluation and rehabilitation of stroke patients. IEEE Trans Neural Systems & Rehab Engng.

[B14] Kahn LE, Zygman ML, Rymer WZ, Reinkensmeyer DJ (2006). Robot-assisted reaching exercise promotes arm movement recovery in chronic hemiparetic stroke: A randomized controlled pilot study. J Neuroengineering and Rehabilitation.

[B15] Johnson MJ, F. VLH, G. BC, Shor P, Leifer LJ (2005). Experimental results using force-feedback cueing in robot-assisted stroke therapy. IEEE Trans Neural Systems & Rehab Engng.

[B16] Emken JL, Reinkensmeyer DJ (2005). Robot-enhanced motor learning: Accelerating internal model formation during locomotion by transient dynamic amplification. IEEE Trans Neural Systems & Rehab Engng.

[B17] Dipietro L, Ferraro M, Palazzolo JJ, Krebs HI, Volpe BT, Hogan N (2005). Customized interactive robotic treatment for stroke: EMG-triggered therapy. IEEE Trans Neural Systems & Rehab Engng.

[B18] Krebs HK, Palazzolo J, Dipietro L, Ferraro M, Krol J, Rannekleiv K, Volpe BT, Hogan N (2003). Rehabilitation Robotics: Performance-based Progressive Robot-Assisted Therapy. Autonomous Robots.

[B19] Jezernik S, Colombo G, Morari M (2004). Automatic gait-pattern adaptation algorithms for rehabilitation with a 4-DOF robotic orthosis. IEEE Trans Robotics and Automation.

[B20] Shadmehr R, Mussa-Ivaldi FA (1994). Adaptive representation of dynamics during learning of a motor task. Journal of Neuroscience.

[B21] Reinkensmeyer DJ, Emken JL, Liu J, Bobrow JE (2004). The Nervous System Appears to Minimize a Weighted Sum of Kinematic Error, Force, and Change in Force when Adapting to Viscous Environments during Reaching and Stepping: Oct; San Diego, CA,..

[B22] Scheidt RA, Dingwell JB, Mussa-Ivaldi FA (2001). Learning to move amid uncertainty. J Neurophysiol.

[B23] Thoroughman KA, Shadmehr R (2000). Learning of action through adaptive combination of motor primitives. Nature.

[B24] Vidyasagar M (1993). Nonlinear Systems analysis.

[B25] Reinkensmeyer DJ (2003). How to Retrain Movement after Neurologic Injury: A Computational Rationale for Incorporating Robot (or Therapist) Assistance. Proceedings of the 2003 IEEE Engineering in Medicine and Biology Society Meeting.

[B26] Emken JL, Wynne JH, Harkema SJ, Reinkensmeyer DJ (2006). A robotic device for manipulating human stepping. IEEE Trans Robotics.

[B27] Caithness G, Osu R, Bays P, Chase H, Klassen J, Kawato M, Wolpert DM, Flanagan JR (2004). Failure to consolidate the consolidation theory of learning for sensorimotor adaptation tasks. J Neurosci.

[B28] Klassen J, Tong C, Flanagan JR (2005). Learning and recall of incremental kinematic and dynamic sensorimotor transformations. Exp Brain Res.

[B29] Kahn LE, Rymer WZ, Reinkensmeyer DJ (2004). Adaptive assistance for guided force training in chronic stroke: September 1-5; San Francisco, California..

[B30] Wool RN, Siegel D, Fine PR (1980). Task performance in spinal cord injury: effects of helplessness training. Arch Phys Med Rehail.

[B31] Grau JW, Barstow DG, Joynes RL (1998). Instrumental learning within the spinal cord: I. Behavioral properties. Behav Neurosci.

[B32] Cai LL, Fong AJ, Otoshi CK, Liang YQ, Cham JG, Zhong H, Roy RR, Edgerton VR, Burdick JW (2005). Effects of consistency vs. variability in robotically controlled training of stepping in adult spinal mice: ; Chicago, IL..

[B33] Kaelin-Lang A, Sawaki L, Cohen LG (2005). Role of voluntary drive in encoding an elementery motor memory. J Neurophysiol.

[B34] Lippman LG, Ress R (1997). Consequences of error production in a perceptual-motor task. J Gen Psychol.

[B35] Kurtzer I, Dizio P, Lackner JR (2003). Task dependent motor learning. Experimental Brain Research.

